# Protective Effect of Daikenchuto on Dextran Sulfate Sodium-Induced Colitis in Mice

**DOI:** 10.1155/2017/1298263

**Published:** 2017-01-22

**Authors:** Takaharu Matsunaga, Shinichi Hashimoto, Naoki Yamamoto, Ryo Kawasato, Tomohiro Shirasawa, Atsushi Goto, Koichi Fujisawa, Taro Takami, Takeshi Okamoto, Jun Nishikawa, Isao Sakaida

**Affiliations:** ^1^Department of Gastroenterology and Hepatology, Yamaguchi University Graduate School of Medicine, 1-1-1 Minami Kogushi, Ube, Yamaguchi 755-8505, Japan; ^2^Yamaguchi University Health Administration Center, 1677-1 Yoshida, Yamaguchi, Yamaguchi 753-8511, Japan; ^3^Center of Research and Education for Regenerative Medicine, Yamaguchi University Graduate School of Medicine, 1-1-1 Minami Kogushi, Ube, Yamaguchi 755-8505, Japan; ^4^Department of Laboratory Science, Yamaguchi University Graduate School of Medicine, 1-1-1 Minami Kogushi, Ube, Yamaguchi 755-8505, Japan

## Abstract

*Aim*. To investigate the effect of daikenchuto (TJ-100; DKT) for ulcerative colitis (UC) model mouse and assess its anti-inflammatory mechanisms.* Methods*. We evaluated the effects of DKT on dextran sulfate sodium- (DSS-) induced experimental colitis. First, we assessed the short-term effects of DKT using two groups: 5% DSS group and 5% DSS with DKT group. Colon length; histological scores; and interleukin- (IL-) 10, IL-1*β*, and tumor necrosis factor-*α* mRNA expression profiles were analyzed using real-time PCR. Second, we assessed the long-term effects of DKT, by comparing survival time between 2% DSS and 2% DSS with DKT groups.* Results*. After 7 days, the colon lengths of DSS + DKT group were longer than those of the DSS group (mean values: 6.11 versus 5.69 cm, *p* < 0.05). Furthermore, compared to DSS group, the DSS + DKT group maintained significantly higher levels of serum hemoglobin (13.1 versus 10.7 g/dL, *p* < 0.05) and exhibited significantly higher expression levels of IL-10 (*p* < 0.05). The 2% DSS + DKT group exhibited significantly longer survival time than the 2% DSS group (70 versus 44 days, *p* < 0.01).* Conclusion*. Our results indicate that DKT prevented inflammation in the colon, indicating its potential as a new therapeutic agent for UC.

## 1. Introduction

Ulcerative colitis (UC) is a chronic, recurrent inflammatory disease of the colon and rectum, characterized by bloody diarrhea, intestinal mucosal ulceration, and infiltration of neutrophils and lymphocytes into the mucous membrane [1]. Although multiple factors such as environmental changes, gene variations, and gut microbiota were thought to be associated with UC, its pathogenesis has not been fully elucidated [2]. In recent years, novel therapeutic agents to treat UC, such as tacrolimus, infliximab, and adalimumab, have become available in Japan, and these new drugs have enabled many patients to avoid surgery [3–5]. However, many of the therapeutic agents used to treat UC are immunosuppressants, and because UC commonly develops in elderly patients, there is a real risk of complications due to infection [6].

Daikenchuto (DKT) is a traditional herbal medicine prepared by mixing Ginseng Radix, Zanthoxyli Fructus, and Zingiberis Processum Rhizoma in a 3 : 2 : 5 ratio, extracting the essence, and then adding Saccharum Granorum. Some studies have reported the clinical efficacy of DKT in treating postoperative paralytic ileus, and recent research has shown that DKT has an inhibitory effect on inflammation in Crohn's disease and prevents colon adhesions in animal models [7–10].

Dextran sulfate sodium (DSS) induces severe mucosal inflammation and colitis, and DSS-induced colitis is considered a type of UC model in terms of morphological and pathophysiological features [11, 12]. DSS-induced colitis model has been generally used to understand the molecular mechanisms of action or to assess the therapeutic effects of test compounds in treatment of UC [13–15].

In this study, we evaluated the effects of DKT on the development of DSS-induced experimental colitis in mice.

## 2. Materials and Methods

### 2.1. Mice

Female C57BL/6 mice, eight weeks of age, with an average weight of 18–20 g were purchased from Chiyoda SLC (Tokyo, Japan). They were acclimatized for 1 week before the experiment and were housed individually in a room maintained at 22°C under a 12 h day/night cycle throughout the experiment. The mice were provided with food and drinking water ad libitum. The mice were maintained in specific pathogen-free housing at the Animal Experiment Facility of Yamaguchi University School of Medicine and cared for in accordance with the animal ethics requirements of the Yamaguchi University School of Medicine.

### 2.2. Assessment of Inflammation in Mouse Model of DSS-Induced Colitis

The mice were divided into three groups (*n* = 10 per group): normal group, DSS group, and DSS + DKT group. Mice in the normal group remained untreated. Mice in the DSS and DSS + DKT groups were both provided with 5% DSS (mol. wt. 5000; Wako Pure Chemical Industries Ltd., Osaka, Japan) through water bottles. Additionally, 6 mg/g body weight of DKT was orally administered using infection tubes in the DSS + DKT group, while 0.2 mL distilled water was orally administered in the same manner in the other two groups. Previous study reported by Case et al. shows that acute oral toxicity study in mice revealed LD50 value 2.64 gm/kg for DSS [16]. Okayasu et al. used 3–10% DSS in their study, and we used 5% DSS for this study [11]. Kono et al. reported anticolitis effect for colitis mouse model using 2.7 mg/g body weight of DKT [10]. According to this study, we used 6 mg/g body weight of DKT which was nearly double to 2.7 mg/g. The intake of both water and food was measured in each group. Serum samples were obtained by eye puncture method on Day 7. In all the experiments, serum hemoglobin (Hb) was measured using an analyzer for clinical chemistry (SPOTCHEM EZ SP-4430; Arkray, Kyoto, Japan). In addition, we recorded the body weight and length of the colon on Day 7.

### 2.3. Histological Assessment

Mice were anesthetized using halothane (Wako, Japan) and killed by cervical dislocation. Histological examination was performed on samples of the distal colon from each mouse. The samples were fixed in 4% formaldehyde overnight at 4°C. Paraffin sections (4 *μ*m) were stained with hematoxylin and eosin (H&E). All histological evaluations were performed according to the histological score calculation method previously described by Morohoshi et al. [15]; the histological score was estimated by the combined of inflammatory cell infiltration and tissue damage. The infiltration scoring was as follows: 0, no infiltration; 1, presence of occasional inflammatory cells in the lamina propria; 2, increased numbers of inflammatory cells in the lamina propria; and 3, confluent inflammatory cells extending into the submucosa. The tissue damage scoring was as follows: 0, no mucosal damage; 1, discrete lymphoepithelial lesions; 2, surface mucosal erosion or focal ulceration; and 3, extensive mucosal damage and extension into deeper structures of the bowel wall. The combined histological score was used for examination. The H&E-stained sections were observed using a Keyence BIOREVO BZ9000 microscope (Osaka, Japan).

### 2.4. Real-Time Quantitative Polymerase Chain Reaction (PCR)

Total RNA was isolated from the distal colons of mice. The mRNA expression profiles of interleukin- (IL-) 10, IL-1*β*, and tumor necrosis factor- (TNF-) *α* were evaluated using real-time quantitative PCR. Briefly, total RNA was extracted using an RNeasy Mini kit (Qiagen GmbH; Hilden, Germany) according to the manufacturer's instructions. The primers used were as follows: mouse IL-10 primers—sense (5′-CCAGTTTTACCTGGTAGAAGTGATG-3′), antisense (5′- TGTCTAGGTCCTGGAGTCCAGCAGACTC-3′); mouse IL-1*β* primers—sense (5′- CACAGCAGCACATCAACAAG-3′), antisense (5′- GTGCTCATGTCCTCATCCTG-3′); mouse TNF-*α* primers—sense (5′- GCCTCTTCTCATTCCTGCTTG-3′), antisense (5′- CTGATGAGAGGGAGGCCATT-3′); and mouse *β*-actin primers—sense (5′- TGACAGGATGCAGAAGGAGA-3′), antisense (5′- GCTGGAAGGTGGACAGTGAG-3′). PCR amplification was performed in triplicate using the following cycle conditions: 40 cycles at 90°C for 30 s, 55–60°C for 45 s, and 72°C for 1 min. *β*-Actin was used as the reference gene.

### 2.5. Survival Time Analysis

To analyze survival time, the mice were divided into two groups: 2% DSS group (*n* = 9) and 2% DSS + DKT (*n* = 9) group, in which the mice were provided 2% DSS or 2% DSS + 3 mg/g body weight DKT, respectively, through water bottles for 100 days.

### 2.6. Statistical Analysis

All values are expressed as the mean ± standard error of the mean (SEM). Statistical significance was determined using two-tailed Student's *t*-test. Differences in *p* values < 0.05 were considered significant. Kaplan–Meier estimator was used for survival time analysis. We used Excel Statistics 2012 (SSRI Co., Ltd.; Tokyo, Japan) for all statistical analyses.

## 3. Results

### 3.1. Morphological and Histological Analyses of DKT-Treated Mice

**Figure 1:**
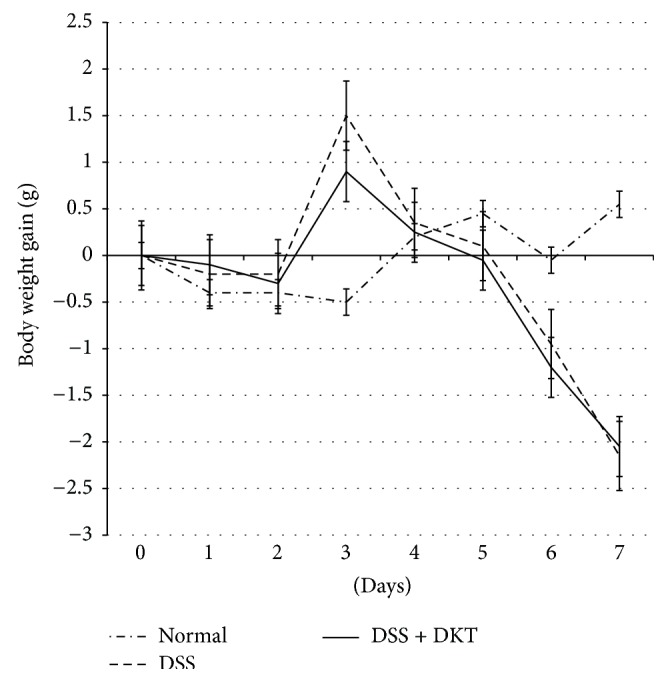
Body weight gain of each study group. Normal group, untreated mice. Dextran sulfate sodium (DSS) group, mice provided with 5% DSS for 7 days. DSS + daikenchuto (DKT) group, mice provided with DKT in addition to DSS. *n* = 10 mice (for normal, DSS, and DSS + DKT groups).

**Figure 2:**
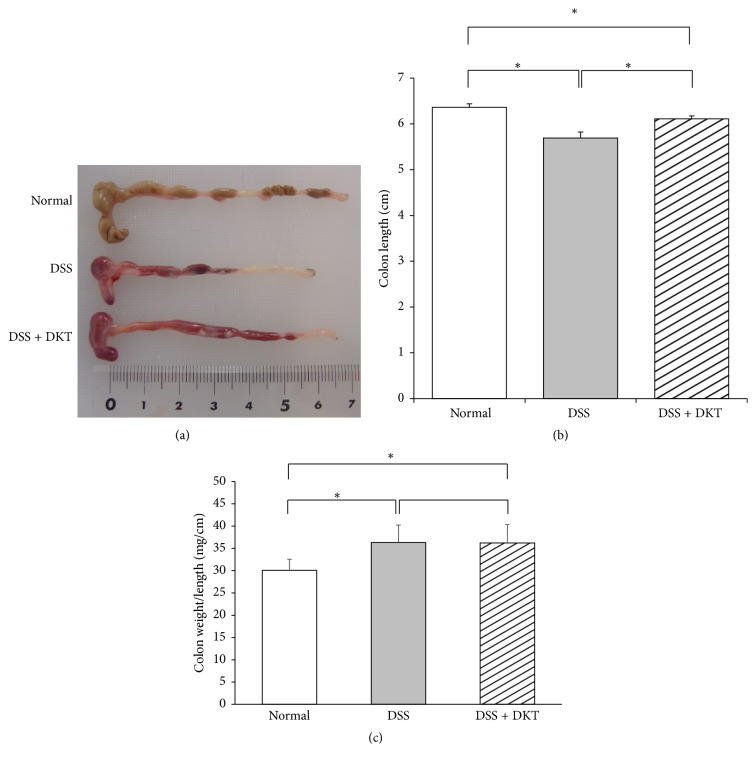
(a) Macroscopic appearance and (b) colon length of each study group. Normal group, untreated mice. Dextran sulfate sodium (DSS) group, mice provided with 5% DSS for 7 days. DSS + daikenchuto (DKT) group, mice provided with DKT in addition to DSS. *n* = 10 mice (for normal, DSS, and DSS + DKT groups). (c) Colon relative weight (weight/length) of each study group.  ^*∗*^*p* < 0.05.

**Figure 3:**
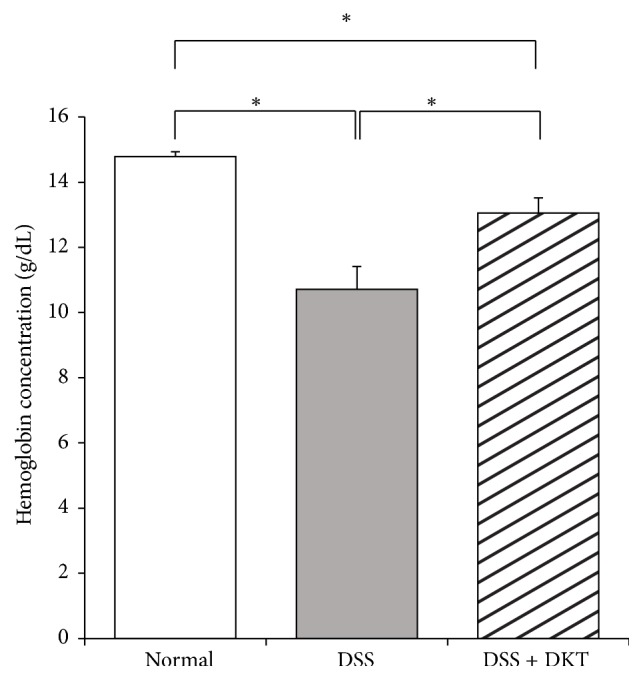
Hemoglobin concentration of each study group. Normal group, untreated mice. Dextran sulfate sodium (DSS) group, mice provided with 5% DSS for 7 days. DSS + daikenchuto (DKT) group, mice provided with DKT in addition to DSS. *n* = 10 mice (for normal, DSS, and DSS + DKT groups).  _  _^*∗*^*p* < 0.05.

**Figure 4:**
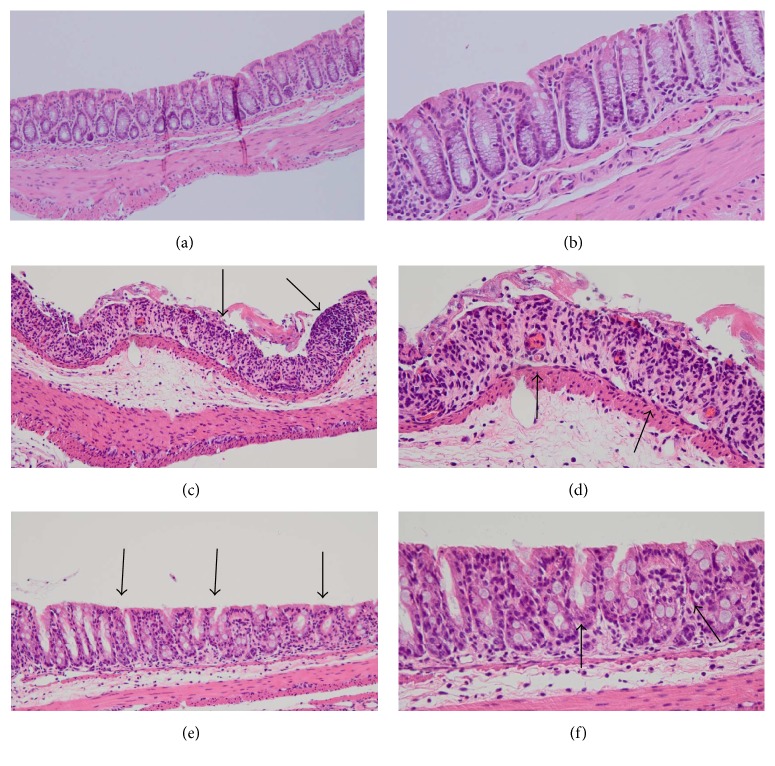
Histopathological analysis of hematoxylin and eosin-stained sections of the distal colon. (a, b) Untreated normal mouse: no disturbance of the ductal structure and no thickening of the wall. (c, d) Mouse provided with 5% dextran sulfate sodium (DSS) for 7 days: disturbance in the ductal structure and thickening of the wall* (arrows)*. (e, f) Mouse administered a combination of 5% DSS and daikenchuto: disturbance in the ductal structure and thickening of the wall, but milder than that in mouse provided with DSS alone* (arrows)*. Original magnification (a), (c), and (e): ×20; (b), (d), and (f): ×40.

**Figure 5:**
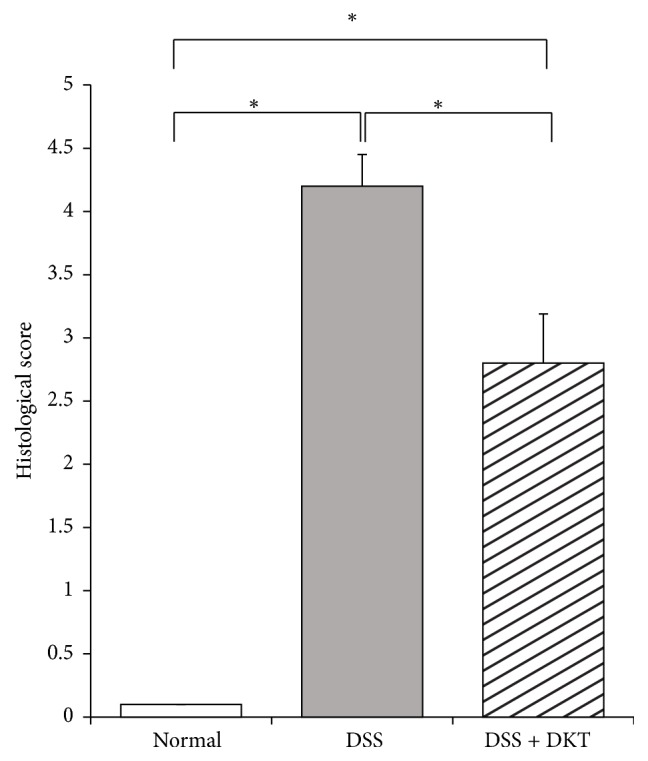
Histological score of each study group. Normal group, untreated mice. Dextran sulfate sodium (DSS) group, mice provided with 5% DSS for 7 days. DSS + daikenchuto (DKT) group, mice provided with DKT in addition to DSS. *n* = 10 mice (for normal, DSS, and DSS + DKT groups). ^*∗*^*p* < 0.05.

As shown in Figure 1, body weight gain of mice on Day 7 was significantly lower in the DSS group and DSS + DKT group than in the normal group (*p* < 0.05). There was no difference between the DSS and the DSS + DKT group in terms of body weight. Morphology analysis of the colon showed that the colon lengths of mice in the DSS + DKT group were significantly longer than those of DSS group mice (*p* < 0.05). Meanwhile, there was no significant difference between DSS + DKT group and DSS group in colon relative weight (weight/length) (Figure 2). The DSS + DKT group showed significantly higher levels of serum Hb than the DSS group (*p* < 0.05) (Figure 3). DSS-induced colitis is characterized by histological findings such as edema, infiltration of inflammatory cells into the mucosa and submucosa, destruction of epithelial cells, and mucosal thickening (Figures 4(a)–4(f)). The histological score was significantly lower in the DSS + DKT group than that in the DSS group (*p* < 0.05) (Figure 5).

### 3.2. Gene Expression in the Colon

**Figure 6:**
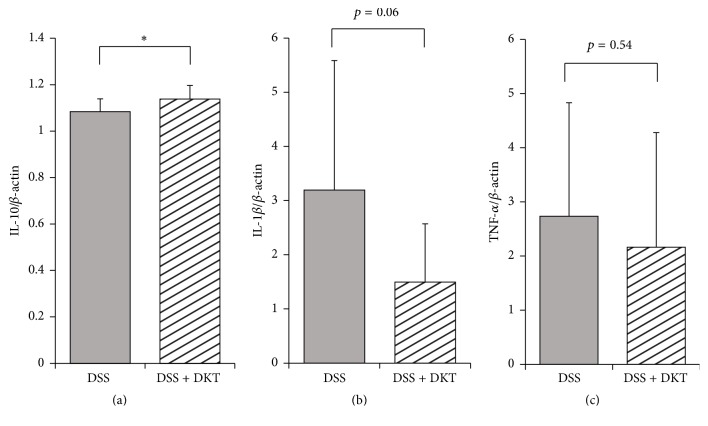
Anti-inflammatory and inflammatory cytokine levels of each study group. (a) Interleukin- (IL-) 10. (b) IL-1*β*. (c) Tumor necrosis factor- (TNF-) *α*. Normal group, untreated mice. Dextran sulfate sodium (DSS) group, mice administered 5% DSS for 7 days. DSS + daikenchuto (DKT) group, mice provided with DKT in addition to DSS. *n* = 10 mice (for normal, DSS, and DSS + DKT groups). ^*∗*^*p* < 0.05.

We analyzed the mRNA expression of IL-10, IL-1*β*, and TNF-*α* in the colon after 7 days of the experiment (Figures 6(a), 6(b), and 6(c)). The IL-10 mRNA expression in the DSS + DKT group was significantly higher than that in the DSS group (*p* < 0.05) (Figure 6(a)). The IL-1*β* and TNF-*α* mRNA expression levels in the DSS + DKT group were lower than those in the DSS group; however, there was no significant difference in the two groups (*p* = 0.06 and 0.54, resp.) (Figures 6(b) and 6(c)).

### 3.3. Survival Time Analysis

**Figure 7:**
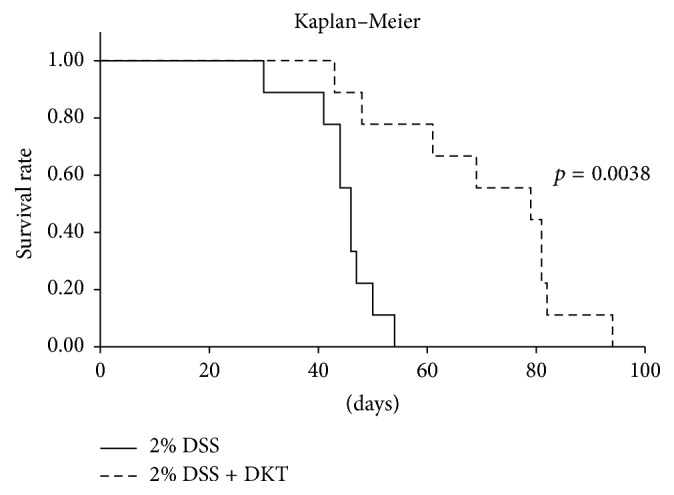
Survival time of each study group. 2% dextran sulfate sodium (DSS) group, mice provided with 2% DSS. 2% DSS + daikenchuto (DKT) group, mice provided with DKT in addition to DSS. *n* = 9 mice (for 2% DSS and 2% DSS + DKT groups).

The survival time in the 2% DSS + DKT group was significantly higher than that in the 2% DSS group (mean value: 70 days versus control 44 days, *p* = 0.0038) (Figure 7).

## 4. Discussion

The purpose of this study was to elucidate the effects of DKT on DSS-induced colitis, and our results showed that DKT attenuated DSS-induced colitis in mice and prolonged the survival of DSS-treated mice. In the DSS group, we noted weight loss, shortening of colon length, and significantly higher histological scores compared with the normal group and thus confirmed that DSS did induce colitis. An associated reduction in peripheral blood Hb concentration was also noted. In the DSS + DKT group, although the weight reduction caused by DSS was not inhibited, the shortening of colon length and elevation of the histological score were significantly inhibited, in addition to a milder reduction in the Hb concentration than that in the DSS group. Furthermore, in the molecular biological investigation via real-time PCR analysis, the IL-10 anti-inflammatory cytokine level was significantly elevated in the DSS + DKT group, while although the difference was not significant, the levels of inflammatory cytokines IL-1*β* and TNF-*α* decreased. This indicates that DKT also exerts an anti-inflammatory effect at the cytokine level.

Kono et al. [10] evaluated the therapeutic effect of DKT using mouse models of colitis induced by intrarectal instillation of 2,4,6-trinitrobenzenesulfonic acid (TNBS); this TNBS-induced colitis model is widely used as Crohn's disease model. The macroscopic and microscopic evaluation scores in their study were lower in the DKT combination group than in the TNBS-only treatment group. The levels of cytokines TNF-*α* and interferon- (INF-) *γ* were lower in the DKT combination group than in the TNBS-only treatment group, and IL-1*β* levels tended to be lower, though not significantly.

Adrenomedullin (ADM) is attracting attention as a central component of the pathway that drives the anti-inflammatory action of DKT. ADM is a peptide belonging to the calcitonin family and is a potent endogenous vasodilator [17]. It is unevenly distributed in the gastrointestinal tract and plays an important role in regulating microcirculation. ADM also exhibits anti-inflammatory action by inhibiting the production of inflammatory cytokines, particularly TNF-*α* [18]. TNF-*α* is an extremely important cytokine in the treatment of Crohn's disease and UC, and investigations have been underway regarding the possibility of using ADM to treat Crohn's disease [19]. In fact, ADM has been found to exert an anti-inflammatory effect in mouse and rat models of Crohn's disease [20, 21]. In a mouse model of DSS-induced colitis, intraperitoneal administration of ADM was shown to inhibit colon inflammation, and research has shown that administration of ADM reduces the levels of cytokines such as TNF-*α*, IL-1*β*, and IL-6 [22]. However, exogenous administration of ADM has not yet been put into practical use because of the necessity to verify the effect of ADM on organs other than those of the gastrointestinal tract and also because ADM does not remain for long at the target site owing to metabolism and clearance [23–25]. Conversely, there have been reports that DKT increases endogenous ADM, which manifests as an anti-inflammatory effect in the colon. In addition, DKT is known to have few adverse reactions and hence may be considered as a method to safely induce ADM expression [10]. We additionally found increased expression of IL-10, but the effect of ADM on IL-10 has not yet been investigated. We therefore consider this information vital for elucidating the action mechanisms of both DKT and ADM.

Furthermore, in this study, in mice administered 2% DSS, it was proven that DKT prolonged survival time. In previous studies of the anti-inflammatory effect in DSS-induced colitis, the majority of the evaluations were conducted over a short period, such as 7 days; however, in this study, we clarified that the action of DKT extends over a long period. In actual clinical practice, a variety of treatments can be used to induce UC remission; however, in Japan, options for remission maintenance therapy for UC comprise only 5-aminosalicylic acid, azathioprine, and anti-TNF-*α* antibodies infliximab and adalimumab. Patients who do not respond adequately to 5-aminosalicylic acid or cannot tolerate azathioprine are administered anti-TNF-*α* antibodies, which are expensive and not always affordable as therapy. In addition, as these drugs exert an immunosuppressant effect, it is essential to be cautious when administering these drugs to elderly patients. DKT is inexpensive, has no immunosuppressant action, and shows long-term protective effects in DSS-induced colitis model, demonstrating its potential for use in remission maintenance therapy for UC.

Previous studies have shown the efficacy of DKT in maintaining postoperative remission in Crohn's disease; however, to the best of our knowledge, no other studies have yet demonstrated the efficacy of DKT in the treatment of inflammatory bowel disease. We believe that the results of our study will promote further research showing the actual efficacy and safety of DKT in treating patients with UC.

The limitations of this study are as follows: we did not confirm the hypothesized anti-inflammatory action mechanism of DKT. It is essential to verify the actual expression of ADM using real-time PCR and immunohistological tests. Further, DKT is a mixture of Ginseng Radix, Zanthoxyli Fructus, and Zingiberis Processum Rhizoma. Although there are several reports about anti-inflammatory effect of Ginseng Radix (Ginsenoside Rb1) and Zingiberis Processum Rhizoma (6-shoganol), the complementary action of the three components has not been elucidated [26, 27].

## 5. Conclusion

To the best of our knowledge, this is the first study to demonstrate the anti-inflammatory effect of DKT in DSS-induced colitis mouse model, which serves as a UC disease model. We also reported the involvement of IL-10 and showed that DKT prolonged the survival time of mice with DSS-induced colitis. We believe that these are important findings for the future development of safe and low-cost novel therapeutic agents for the treatment of UC.
